# Mentorship Quality and Leadership Development in Saudi Nursing Education: A Cross-Sectional Analysis

**DOI:** 10.3390/nursrep16010013

**Published:** 2026-01-05

**Authors:** Ibrahim Alenezi, Fathia Ahmed Mersal, Faisal Khalaf Alanazi

**Affiliations:** 1 Public Health Nursing Department, College of Nursing, Northern Border University, Arar 73213, Saudi Arabia; in.alenezi@nbu.edu.sa; 2 Medical Surgical Nursing Department, College of Nursing, Northern Border University, Arar 73213, Saudi Arabia; faisal.alanazi@nbu.edu.sa

**Keywords:** clinical mentorship, leadership competencies, nursing students, nursing education, Saudi Arabia

## Abstract

**Background:** The healthcare industry demands nurses with both clinical proficiency and leadership skills. However, formal leadership training remains limited among undergraduate nursing students, underscoring the need for clinical mentorship to support leadership development. **Purpose:** This study investigated the association between clinical mentorship quality and leadership competencies among undergraduate nursing students enrolled at a public university in northern Saudi Arabia. **Methods:** Data were collected using a cross-sectional design from 224 nursing students through a self-administered online survey, which comprised three sections: demographic information, students’ perceptions of clinical mentorship quality, and a standardized assessment of leadership competencies. **Results:** Students reported positive perceptions of their leadership competencies, with an average score of 2.82. A strong positive correlation was observed between mentorship quality and leadership competencies, particularly in strategic thinking, emotional intelligence, influence, and teamwork. Differences were observed based on sex and academic performance, with female students and those with higher GPAs exhibiting stronger leadership competencies. Regression analysis revealed mentorship quality (*β* = 0.642, *p* < 0.001) and academic performance (*β* = 0.131, *p* = 0.013) as significant predictors of leadership competencies, while gender and academic year were not statistically significant. **Conclusions:** High-quality clinical mentorship substantially contributes to the development of leadership competencies among nursing students, with academic performance providing additional support. Integrating structured mentorship programs into nursing curricula may enhance preparedness for leadership roles within healthcare settings.

## 1. Introduction

Contemporary healthcare systems require nursing professionals who combine clinical expertise with transformational leadership capabilities [1]. Traditional nursing education has emphasized technical competencies, yet modern healthcare environments demand nurses capable of navigating complex organizational structures, leading interdisciplinary teams, and driving quality improvement initiatives [2]. A persistent theory–practice gap exists between academic preparation and the leadership competencies required in professional settings [3]. Students often graduate with strong clinical skills but limited confidence in leadership roles [4], with consequences extending beyond individual career development to affect patient outcomes, healthcare quality, and organizational effectiveness [5]. Understanding how educational interventions, particularly clinical mentorship, can foster leadership development among undergraduate nursing students has therefore become urgent.

Contemporary healthcare demands strategic thinking, emotional intelligence, collaborative influence, and team coordination, competencies often inadequately addressed in conventional nursing curricula [6]. Early-career nurses increasingly assume leadership responsibilities within complex healthcare teams despite limited formal preparation during undergraduate education. In Saudi Arabia, Vision 2030 has positioned healthcare transformation as a national priority, recognizing nursing as essential to interdisciplinary healthcare delivery [7]. Realizing these ambitious goals requires nursing graduates who possess both clinical competencies and the leadership capabilities necessary to drive organizational change and innovation.

Clinical mentorship has emerged as a powerful mechanism for bridging the gap between academic preparation and professional leadership requirements. Effective mentorship provides students with opportunities to observe, practice, and internalize leadership behaviors within authentic clinical contexts [8]. High-quality mentorship experiences contribute to enhanced communication skills, increased self-confidence, improved problem-solving abilities, and greater professional commitment [9]. Mentorship relationships also provide access to professional networks, career guidance, and skill refinement opportunities that extend beyond formal curricula [10]. However, the specific mechanisms through which mentorship quality influences leadership competency development remain underexplored, particularly in Middle Eastern contexts where cultural factors may significantly shape mentor–student relationships.

This study adopts an integrated theoretical framework combining Transformational Leadership Theory and Social Learning Theory to explore the relationship between clinical mentorship quality and leadership competency development. Transformational Leadership Theory, initially conceptualized by Burns [11] and later refined by Bass and Avolio [12], delineates four core dimensions: idealized influence (role modeling), inspirational motivation (articulating compelling visions), intellectual stimulation (promoting innovation), and individualized consideration (providing tailored support). Within clinical mentorship contexts, these behaviors manifest through practices that foster leadership growth, such as modeling professional integrity, inspiring students to envision their contributions to nursing, encouraging critical inquiry, and offering personalized guidance [13,14].

Social Learning Theory [15] complements this perspective by elucidating the cognitive and behavioral mechanisms underpinning leadership acquisition. It posits that individuals learn complex behaviors through observation, modeling, and vicarious reinforcement within social environments. In clinical education, students develop leadership competencies by observing mentors who exemplify effective leadership in authentic healthcare settings [16]. Repeated exposure enables internalization of decision-making strategies and interpersonal skills, while enhanced self-efficacy, central to Bandura’s framework, strengthens confidence in performing leadership roles.

Integrating these theories suggests that high-quality mentorship optimizes leadership development by combining transformational modeling with structured observational learning [17]. When mentors embody transformational principles and provide guided exposure to leadership situations, they facilitate both skill acquisition and self-efficacy. This approach underscores leadership development as a dual cognitive behavioral process requiring authentic practice contexts, expert modeling, and individualized support.

Despite theoretical foundations supporting mentorship-based leadership development, significant empirical gaps remain, particularly within undergraduate nursing education contexts. While existing research has examined mentorship effects on clinical competencies and general professional development, limited investigation exists regarding the specific mechanisms through which mentorship quality influences leadership competency acquisition among nursing students. This knowledge gap is particularly pronounced in Middle Eastern educational contexts, where cultural factors, educational traditions, and healthcare system characteristics may influence mentorship relationships and leadership development pathways differently than in Western settings. The absence of empirical evidence from these contexts limits our ability to develop culturally responsive mentorship programs aligned with regional healthcare transformation initiatives such as Saudi Vision 2030.

Most undergraduate nursing programs lack systematic approaches to leadership competency cultivation despite its recognized importance. This study addresses these gaps by providing the first comprehensive quantitative analysis of mentorship–leadership relationships within Saudi Arabian nursing education, offering insights to inform evidence-based program development and educational policy decisions. The significance extends beyond academic inquiry to encompass practical implications for healthcare workforce development, patient care quality, and healthcare system transformation. By identifying the specific mentorship characteristics that most effectively promote leadership development, this study can inform the design of structured mentorship programs that better prepare nursing graduates for professional leadership challenges.

Based on the integrated theoretical framework and existing literature, this study hypothesizes that clinical mentorship quality demonstrates a significant positive association with leadership competency development among undergraduate nursing students. Specifically, students who report higher levels of mentorship quality will demonstrate stronger leadership competencies across the domains of strategic thinking, emotional intelligence, impact and influence, and teamwork skills, after controlling for relevant demographic and academic variables.

This study examines how clinical mentorship quality influences leadership competency development among undergraduate nursing students in Saudi Arabia. It explores associations with four leadership domains, strategic thinking, emotional intelligence, impact and influence, and teamwork, while considering demographic and academic moderators and potential gender differences. The findings aim to inform structured mentorship programs that strengthen leadership in nursing education.

## 2. Methods

### 2.1. Research Design and Setting

This descriptive cross-sectional study examined the relationship between clinical mentorship quality and leadership competency development among undergraduate nursing students at Northern Border University, a public institution in northern Saudi Arabia. The cross-sectional design enabled simultaneous assessment of multiple variables at a single time point, providing efficient data collection within academic scheduling constraints while offering insights into current mentorship–leadership relationships [17]. The study setting represents a typical Saudi nursing education environment where students engage in theoretical coursework and supervised clinical rotations across various healthcare facilities, providing authentic mentorship experiences relevant to Saudi Vision 2030 healthcare transformation initiatives.

### 2.2. Sampling Strategy

The target population comprised 380 undergraduate nursing students enrolled in years 2–4 of the nursing program. Sample size calculation using G*Power 3.1.9.7 software was based on detecting a medium effect size (r = 0.30) with α = 0.05 and power of 0.80, yielding a minimum requirement of 84 participants [18]. To accommodate multiple regression analysis with five predictors, potential non-response, and subgroup analyses, the target sample was increased to 224 participants, providing 99% power for medium effects (r = 0.30) and 85% power for small-to-medium effects (r = 0.25).

Convenience sampling was employed based on methodological, practical, and contextual considerations. As the first quantitative investigation of mentorship–leadership relationships in Saudi nursing education, this approach was appropriate for initial hypothesis generation [19]. The target population demonstrated relative homogeneity in educational structure, cultural background, and professional preparation, reducing typical heterogeneity concerns. Practical constraints included intricate student rotation schedules across multiple clinical sites and examination periods, making random sampling logistically challenging. Additionally, convenience sampling aligned with Saudi cultural values emphasizing voluntary participation and respect for personal autonomy.

### 2.3. Bias Mitigation Strategies

Several potential biases were systematically addressed. Selection bias was mitigated through diversified recruitment across clinical rotations, theoretical classes, and informal gatherings over three months, capturing varied engagement levels and perspectives. The high response rate (87.5% of approached students) and demographic representativeness across academic years, gender, and performance ranges provided evidence of reasonable sample representativeness. Volunteer bias was addressed through recruitment messaging emphasizing the value of diverse perspectives, including negative experiences, with assurances that all viewpoints would contribute meaningfully to nursing education improvement. Academic performance bias was minimized through stratified recruitment targeting students across the full GPAs spectrum, with materials emphasizing equal value regardless of academic standing.

Social desirability bias was addressed through strict anonymity maintenance, explicit statements that honest responses were most valuable, and frequency-based behavioral items focusing on observable behaviors rather than self-evaluative judgments. Response bias related to survey fatigue was minimized through optimized survey design, including concise item wording, logical organization with progress indicators, mobile optimization, and pilot testing, ensuring a 15–20 min completion time. Quality control procedures identified and excluded responses with suspicious patterns, including straight-lining or implausibly rapid completion.

### 2.4. External Validity and Generalizability

Several limitations regarding external validity must be acknowledged. Findings are derived from a single institution in northern Saudi Arabia, potentially limiting transferability due to institution-specific factors such as curriculum design, clinical partnerships, and student demographics. Regional factors, including healthcare infrastructure and cultural norms, may influence mentorship–leadership pathways differently than other contexts. Sample homogeneity, while reducing within-sample variance, limits generalizability to more diverse populations and institutions with different curricular approaches. The four-month data collection period captured a specific temporal snapshot, precluding assessment of seasonal variations or developmental trajectories.

Future research should address these limitations through probability sampling approaches (e.g., stratified random sampling), multi-site studies encompassing diverse institutions and regions, longitudinal designs following students across academic years, and mixed-methods approaches combining quantitative surveys with qualitative interviews to provide a mechanistic understanding and contextual nuance.

### 2.5. Participant Selection

Of 380 eligible students, 256 were systematically approached through multi-phase recruitment involving direct classroom presentations, clinical site coordination with instructors, and digital outreach through official university channels. Thirty-two students declined participation due to time constraints (n = 18), lack of interest (n = 8), or anonymity concerns (n = 6), yielding a final sample of 224 participants (59% of the eligible population; 87.5% of approached students).

Inclusion criteria required current enrollment in years 2–4, completion of at least one supervised clinical rotation with mentor interaction, and voluntary informed consent. Exclusion criteria included first-year status without clinical mentorship experience, non-enrollment in the undergraduate program, or unavailability during data collection.

### 2.6. Instrumentation

Data collection utilized a comprehensive electronic survey via Google Forms, incorporating three sections:

Demographic section collected age (<20 years, ≥20 years), gender, marital status, academic year, and GPAs (≤3.5, >3.5), selected based on literature suggesting potential influences on mentorship experiences and leadership development [20,21].

Mentorship quality assessment employed a validated 12-item questionnaire [22] with 6-point Likert scales (0 = strongly disagree to 5 = strongly agree), assessing five dimensions: mentor availability and accessibility, professional support and guidance, content expertise and knowledge sharing, feedback quality and constructive critique, and professional development and networking. Because English is the primary language of nursing education in Saudi Arabia, the questionnaire was provided in both English and Arabic. As no validated Arabic version was available, the authors conducted forward and backward translations by bilingual experts, expert panel review for cultural relevance, and pilot testing with undergraduate nursing students to confirm clarity and reliability.

Leadership competency assessment employed a 40-item questionnaire synthesizing established frameworks [6,23] with contemporary nursing leadership models. Items used 5-point frequency-based Likert scales (0 = never to 4 = almost always), assessing four domains: strategic thinking (10 items), emotional intelligence (10 items), impact and influence (10 items), and teamwork skills (10 items). This frequency-based approach focuses on behavioral manifestations rather than self-perceptions [24].

For both instruments, composite scores were calculated by summing items within domains, converting to percentage scores [(Actual Score ÷ Maximum Possible Score) × 100], and calculating weighted averages. 75% threshold dichotomized responses into positive/good (≥75%) and negative/poor (<75%) categories, reflecting high-performance expectations in healthcare education [25].

### 2.7. Psychometric Properties

Both instruments demonstrated exceptional internal consistency: leadership competency assessment (Cronbach’s α = 0.990) and mentorship quality assessment (α = 0.985). Individual leadership domains also showed strong reliability: strategic thinking (α = 0.92), emotional intelligence (α = 0.94), impact and influence (α = 0.91), and teamwork skills (α = 0.93), all exceeding acceptable thresholds (α > 0.70; [26]).

Content validity was enhanced through expert panel review involving a senior nursing faculty member (15+ years’ experience), a healthcare leadership consultant, and a psychometrician specializing in Arabic instrument adaptation. The review process achieved Content Validity Index scores > 0.85 for all items. Strong positive correlations between mentorship quality and leadership competency domains (r = 0.57–0.63, all *p* < 0.001) provided preliminary evidence for convergent validity.

### 2.8. Data Collection Procedures

Google Forms were configured with mandatory field settings, built-in consistency checks, progress indicators, and mobile optimization. Data collection occurred from September 20 through 31 December 2024, with daily response monitoring, weekly quality checks, and immediate technical issue management. Quality assurance measures included response completeness requirements (systematic exclusion of responses with >20% missing data, affecting <2% of submissions), response pattern analysis (detecting straight-lining and minimal variance), and logical consistency validation (cross-item consistency checks).

Data security measures encompassed password-protected storage with restricted access, encrypted transmission, complete anonymization with no personally identifiable information, IP address masking, and aggregate-only reporting.

### 2.9. Ethical Considerations

Comprehensive ethical approval was obtained from the Local Committee of Bioethics at Northern Border University (Approval Reference: HAP-09-A-043, approval number 102/24/H, dated 16 September 2024), with formal authorization from the college dean. Electronic informed consent included a clear explanation of the study purpose, participation requirements, voluntary nature, anonymity protections, and researchers’ contact information. Participants provided explicit consent through reading confirmation checkboxes and a final comprehensive consent checkbox with digital timestamp recording.

Complete anonymity (rather than confidentiality) was maintained through survey configuration, preventing the collection of identifying information, IP address masking, and aggregate-only data presentation with minimum cell size requirements. Risk minimization focused on assurances that responses would not affect academic standing or mentor relationships, with time burden minimized through survey optimization for 15–20 min completion.

### 2.10. Statistical Analysis

All analyses were conducted using SPSS version 28.0 with supplementary analyses in R version 4.3.0. Descriptive statistics characterized participant demographics through frequencies, percentages, means, standard deviations, medians, and interquartile ranges, with normality assessed via Shapiro–Wilk tests. Bivariate analyses employed Mann–Whitney U tests for two-group comparisons and Kruskal–Wallis H tests for multi-group comparisons, selected due to non-normal distributions. Spearman’s rank correlation coefficient assessed continuous relationships.

Multiple linear regression evaluated predictors of leadership competencies:

Leadership Competencies = β_0_ + β_1_; (Mentorship Quality) + β_2_ (Gender) + β_3_ (Academic Year) + β_4_ (GPA) + ε

Bootstrap resampling with 5000 samples generated bias-corrected and accelerated (BCa) confidence intervals, reducing dependence on normality assumptions. Model evaluation included F-statistical significance, adjusted R^2^, *t*-tests with bootstrap confidence intervals, residual analysis, and variance inflation factors for multicollinearity assessment.

Statistical assumptions were systematically tested: normality (Shapiro–Wilk tests, Q-Q plots), linearity (scatterplots, residual analysis), homoscedasticity (Levene’s tests), and independence (Durbin–Watson statistics). With minimal missing data (<2%), listwise deletion was employed with sensitivity analyses confirming minimal impact. Statistical significance was set at α = 0.05 with 95% confidence intervals reported. Bonferroni correction was applied for multiple post hoc comparisons to control familywise error rates.

## 3. Results

The study recruited 224 participants from 380 eligible undergraduate nursing students at Northern Border University (59% of the eligible population). Of 256 students approached across multiple recruitment phases, 224 provided complete responses (87.5% response rate). Thirty-two students declined participation, citing time constraints (n = 18, 56.3%), lack of research interest (n = 8, 25.0%), and confidentiality concerns (n = 6, 18.7%). The final sample demonstrated strong representativeness across key demographic variables (Table 1). Age distribution showed 135 participants (60.3%) under 20 years, reflecting typical Saudi undergraduate nursing program demographics. Gender distribution was nearly balanced: 115 females (51.3%) and 109 males (48.7%). The overwhelming majority (99.1%, n = 222) were unmarried, consistent with traditional undergraduate populations in the regional context.

Academic year distribution reflected natural enrollment patterns: second-year students (n = 39, 17.4%), third-year students (n = 112, 50.0%), and fourth-year students (n = 73, 32.6%). Academic performance distribution showed 172 participants (76.8%) with GPAs > 3.5, indicating a high-achieving sample reflecting competitive nursing program admission standards at Northern Border University. The multi-phase recruitment approach (classroom, clinical site, and digital outreach) successfully minimized anticipated biases. The extended three-month data collection period with multiple recruitment waves reduced temporal response bias, while stratified recruitment across diverse settings captured varied engagement levels and academic performance ranges (Table 1).

Both measurement instruments demonstrated exceptional psychometric properties. The leadership competency assessment achieved outstanding internal consistency (Cronbach’s α = 0.990), with individual domains showing strong reliability: strategic thinking (α = 0.92), emotional intelligence (α = 0.94), impact and influence (α = 0.91), and teamwork skills (α = 0.93). The clinical mentorship quality assessment similarly demonstrated exceptional consistency (α = 0.985), substantially exceeding conventional acceptability thresholds. Comprehensive quality assurance measures ensured data integrity throughout collection. Response completeness was achieved for 98% of submissions, with systematic exclusion of responses with >20% missing data affecting fewer than 2% of submissions. Response pattern analysis identified no evidence of systematic straight-lining or unusually rapid completion times.

Mentorship Quality Perceptions. Students reported generally positive mentorship experiences with an overall effectiveness mean of 3.43 (SD = 1.34) on the 6-point scale, with 169 participants (75.4%) expressing agreement or strong agreement regarding mentorship quality (Table 2). The highest-rated dimension was resource suggestion and networking support (M = 3.49, SD = 1.411), with 174 participants (77.7%) expressing agreement. Demonstration of content expertise emerged as the highest-rated dimension (M = 3.50, SD = 1.398), with 172 participants (76.8%) reporting positive experiences, indicating that students particularly value mentors’ instrumental support and professional competence during clinical rotations. Professional guidance dimensions consistently received strong ratings: supportiveness and encouragement (75.9% agreement), responsiveness to questions (75.9% agreement), and professional guidance on networking (75.9% agreement). However, motivational support for performance improvement emerged as the lowest-rated dimension (M = 3.37, SD = 1.488), with 161 participants (71.9%) expressing agreement, representing the only mentorship dimension falling below the 75% agreement threshold and indicating a significant opportunity for program enhancement.

Leadership Competency Assessment. Participants demonstrated moderate leadership confidence across all assessed domains, with a total competency score of 2.82 (SD = 1.00) on the 5-point frequency scale. Using the established 75% threshold (score ≥ 3.75), 145 participants (64.7%) reported positive leadership self-perceptions, while 79 participants (35.3%) reported negative self-assessments (Table 3 and Supplementary Materials). Leadership competencies showed relatively balanced development across all four domains. Emotional intelligence demonstrated the highest mean score (M = 2.84, SD = 1.00), with 146 participants (65.2%) reporting positive self-perceptions. Teamwork skills (M = 2.82, SD = 1.03, 63.8% positive) and strategic thinking (M = 2.81, SD = 1.01, 62.9% positive) showed comparable levels. Impact and influence competency demonstrated similar mean scores (M = 2.80, SD = 1.01) but the highest percentage of positive self-perceptions (65.6%), indicating strong student confidence in their ability to influence others and drive change. The substantial proportion of students reporting negative leadership self-perceptions (35.3%) warrants attention, suggesting that current educational approaches may not effectively build leadership confidence across all students.

Mentorship-Leadership Correlations. Spearman’s rank correlation analysis revealed strong positive associations between mentorship quality and all leadership competency domains (Table 4). All correlations achieved statistical significance at *p* < 0.001 (two-tailed), with correlation coefficients ranging from ρ = 0.570 to ρ = 0.625. Teamwork skills demonstrated the strongest correlation with mentorship quality (ρ = 0.625, *p* < 0.001), followed closely by emotional intelligence (ρ = 0.624, *p* < 0.001), suggesting that relational and interpersonal aspects of mentorship may be particularly influential in developing collaborative and emotionally intelligent leadership behaviors. Strategic thinking showed a strong correlation (ρ = 0.607, *p* < 0.001), while impact and influence demonstrated a moderate-to-strong relationship (ρ = 0.570, *p* < 0.001). The overall leadership competency score showed a strong positive correlation with mentorship quality (ρ = 0.618, *p* < 0.001), indicating that students experiencing higher-quality mentorship relationships consistently report greater leadership confidence across all domains.

Demographic Influences. Significant gender differences emerged in both mentorship quality perceptions and leadership competencies (Table 5). Female students reported significantly higher mentorship quality scores (M = 3.84, SD = 1.16) compared to male students (M = 3.01, SD = 1.39; z = −4.467, *p* < 0.001), representing a medium-to-large effect size (Cohen’s d = 0.65). Female students also demonstrated significantly higher leadership competency scores (M = 3.03, SD = 1.04) compared to male students (M = 2.60, SD = 0.91; z = −3.956, *p* < 0.001), representing a medium effect size (Cohen’s d = 0.45). Students with GPAs > 3.5 demonstrated significantly higher leadership competency scores (M = 2.91, SD = 0.97) compared to those with GPAs ≤ 3.5 (M = 2.51, SD = 1.02; z = −2.510, *p* = 0.012), representing a medium effect size (Cohen’s d = 0.40). Importantly, mentorship quality perceptions showed no significant differences by academic performance (*p* = 0.398), indicating that mentorship accessibility and quality remain equitable across achievement levels. Leadership competencies showed no significant variation across academic years (*p* = 0.249), challenging assumptions about linear leadership development through educational progression. However, mentorship quality perceptions varied significantly, with second-year students reporting higher satisfaction (M = 3.88, SD = 1.49) compared to third-year (M = 3.47, SD = 1.31) and fourth-year students (M = 3.14, SD = 1.24; Z = 11.092, *p* = 0.004), suggesting potential deterioration in mentorship effectiveness as students’ progress.

Predictive Modeling. Multiple linear regression analysis with bootstrap resampling (5000 samples) examined the predictive relationships between demographic variables, mentorship quality, and leadership competencies (Table 6). The overall model was statistically significant (F = 38.089, *p* < 0.001) and explained substantial variance in leadership competencies (R^2^ = 0.411, adjusted R^2^ = 0.399), indicating that the included predictors account for approximately 40% of leadership competency variation. Mentorship quality emerged as the strongest predictor of leadership competency development. The unstandardized coefficient was B = 0.478 (95% CI: 0.382–0.570), indicating that each 1-unit increase in mentorship effectiveness corresponds to a 0.478-unit increase in leadership competency when controlling for demographic variables. The standardized coefficient was β = 0.642, *p* < 0.001, confirming a strong positive effect. Academic performance (GPA) also showed a significant but more modest effect (B = 0.202, 95% CI: 0.043–0.366; β = 0.131, *p* = 0.014). Neither gender (β = −0.002, *p* = 0.968) nor academic year (β = 0.088, *p* = 0.104) was a significant predictor after controlling for mentorship quality and GPAs, suggesting that observed bivariate gender differences may be mediated through variations in mentorship experiences rather than direct gender effects.

## 4. Discussion

The robust predictive relationship between mentorship quality and leadership competencies (β = 0.642, *p* < 0.001) provides compelling empirical validation for the integrated theoretical framework combining Social Learning Theory and Transformational Leadership Theory within Saudi nursing education. Correlation patterns across leadership domains, with teamwork competencies (ρ = 0.625) and emotional intelligence (ρ = 0.624) showing the strongest relationships, suggest that effective mentorship primarily facilitates leadership development through social learning processes and modeling of interpersonal behaviors [15,27]. The differential correlation strengths offer valuable mechanistic insights: the particularly strong relationships with teamwork and emotional intelligence indicate that clinical mentorship provides rich opportunities for observing and practicing collaborative leadership behaviors, with mentors serving as proximal role models during patient care interactions. The somewhat lower correlations with strategic thinking (ρ = 0.607) and impact and influence (ρ = 0.570) suggest that cognitive and influencing competencies may require complementary developmental approaches beyond traditional mentorship, such as case-based learning, leadership simulations, and formal curricular integration [28].

Transformational Leadership Theory explains this relationship through mentors’ demonstration of idealized influence, inspirational motivation, intellectual stimulation, and individualized consideration. These transformational behaviors create the optimal social context for Social Learning Theory’s mechanisms, observation, modeling, and vicarious reinforcement, enabling students to internalize mentors’ leadership behaviors and decision-making processes. This synergistic process is further reinforced through leadership self-efficacy development, as individualized consideration builds students’ confidence to apply observed competencies in practice.

The pronounced gender differences in both mentorship quality perceptions and leadership competencies represent a significant and concerning finding requiring immediate attention. Female students’ substantially higher mentorship satisfaction (M = 3.84 vs. M = 3.01, Cohen’s d = 0.65) and leadership competency scores (M = 3.03 vs. M = 2.60, Cohen’s d = 0.45) suggest systematic rather than incidental differences. These disparities may reflect the intersection of traditional mentorship communication styles with Saudi cultural contexts, where female students may exhibit distinct help-seeking behaviors, relationship-building preferences, and communication patterns that align more closely with conventional mentorship approaches [29]. Additionally, nursing’s historical emphasis on interpersonal relationships and collaborative care may inadvertently privilege leadership styles more typical of female students, potentially limiting recognition of alternative approaches favored by male students [30].

The consistency of gender effects across both mentorship experiences and leadership outcomes suggests current educational structures may not provide equitable developmental opportunities. This imbalance threatens broader patterns of gender representation in nursing leadership roles, particularly given Saudi Vision 2030’s emphasis on inclusive leadership development and gender equity in healthcare professional advancement [31]. These findings necessitate differentiated mentorship approaches accommodating diverse communication styles, learning preferences, and leadership pathways. For male students, this may include peer mentorship models emphasizing collaborative learning, project-based leadership opportunities focused on technical competence and innovation, and mentorship balancing interpersonal skill development with achievement-oriented professional growth. Mentor training programs should incorporate cultural competency and gender-responsive strategies to ensure inclusive engagement across all student demographics [28,32].

The significant association between academic performance and leadership competencies (β = 0.131, *p* = 0.014), independent of mentorship quality, provides important insights into leadership development’s cognitive foundations. High-achieving students often demonstrate advanced metacognitive awareness, self-regulation capabilities, and strategic learning approaches that translate to effective leadership practice [33]. The absence of significant academic performance differences in mentorship quality perceptions, alongside GPA’s independent predictive relationship with leadership competencies, suggests academic achievement may function as a moderator rather than a mediator of mentorship effectiveness. High-achieving students’ enhanced cognitive engagement and metacognitive awareness enable them to extract, synthesize, and apply leadership insights more effectively during clinical encounters [34].

These findings highlight the importance of targeted support across the academic performance spectrum. While high-achieving students may naturally maximize mentorship benefits, students with lower academic performance may require more structured guidance connecting theoretical leadership concepts with practical mentorship experiences through reflective journaling, structured leadership portfolios, or peer learning partnerships [35,36].

The lack of significant leadership competency differences across academic years, despite increased clinical exposure and theoretical instruction, challenges assumptions that leadership naturally evolves through traditional nursing education. Leadership development requires deliberate, longitudinal interventions tracking behavioral, cognitive, and status-related changes over time rather than passive accumulation of experience [37]. The significant decline in mentorship quality perceptions from the second year (M = 3.88) to the third year (M = 3.47) and fourth year (M = 3.14) represents a troubling pattern warranting immediate investigation. This downward trajectory may reflect rising student expectations as clinical knowledge increases, reduced mentor availability during specialized rotations, and structural shifts toward more task-oriented and less relationship-focused mentorship in advanced settings. Mentorship functions as a complex adaptive system shaped by evolving goals, personalized needs, and contextual dynamics; its quality deteriorates when systems fail to adapt to these changing demands [38].

These findings necessitate a fundamental reconsideration of leadership development integration throughout nursing education. Rather than assuming natural progression, curricula should incorporate explicit developmental milestones, structured reflection activities, and progressive mentorship models, maintaining relationship quality while adapting to students’ evolving needs. Leadership development requires tailored approaches reflecting increasing complexity in learners’ understanding and roles rather than one-size-fits-all models [39].

## 5. Limitations

Several limitations warrant acknowledgment. First, a cross-sectional design prevents causal inference between mentorship quality and leadership competencies; longitudinal studies are needed to track developmental trajectories. Second, exclusive reliance on self-report measures introduces potential common method bias and social desirability bias, possibly inflating observed relationships. Third, convenience sampling from a single northern Saudi Arabian institution limits generalizability, particularly to private institutions or other regions. While justified by the study’s exploratory nature and practical constraints, findings are most applicable to similar academic healthcare settings. The high-achieving academic profile (76.8% with GPAs >3.5) may further limit generalizability to more academically diverse populations, though this reflects competitive nursing admission standards.

Fourth, potential biases include selection bias (voluntary participation may reflect more motivated students), researcher bias (minimized through standardized instruments and anonymous collection), and participant bias (social desirability possibly inflating self-reports, partially mitigated through anonymity emphasis). Finally, while the survey was administered via clearly labeled sections, the combined 50+ items may have introduced cognitive fatigue or satisficing behaviors, particularly for participants completing surveys under time constraints or on mobile devices. Quality-control measures detected no strong evidence of such bias, but this possibility cannot be entirely ruled out.

Future research should employ longitudinal designs tracking mentorship–leadership relationships from education into early career stages, revealing critical transitions and optimal intervention timing. Randomized controlled trials comparing traditional and gender-responsive mentorship approaches can identify best practices. Multi-source assessments incorporating peer, supervisor, and behavioral data should reduce bias and improve validity, while qualitative insights from students and mentors can inform culturally relevant mentorship design.

## 6. Conclusions and Recommendations

This study provides robust empirical evidence for mentorship’s critical role in fostering leadership competencies among undergraduate nursing students while revealing significant demographic disparities and developmental challenges demanding immediate attention. High-quality mentorship serves as the primary catalyst for leadership development, accounting for substantial outcome variance beyond demographic and academic factors.

However, pronounced gender differences in both mentorship experiences and leadership outcomes, coupled with declining mentorship quality across academic progression, highlight systemic challenges threatening educational equity and effectiveness. These findings call for immediate action in developing gender-responsive mentorship approaches, implementing structured leadership development curricula, and establishing continuous quality improvement systems for mentorship programs.

Implications extend beyond individual educational experiences to broader healthcare transformation initiatives, making this work particularly relevant for Saudi Vision 2030 objectives. Nursing education programs must prioritize evidence-based mentorship enhancement and integrated leadership development to prepare future nurse leaders capable of driving transformational change in increasingly complex healthcare environments.

Specific recommendations include:

Integrate structured mentorship models: Nursing colleges should formally implement transformational mentorship models, emphasizing mentors’ roles in actively modeling and facilitating the four key leadership competency domains identified in this study.

Implement mandatory mentor training: All clinical mentors should undergo standardized training focused on leadership development best practices, covering Transformational Leadership Theory and Social Learning Theory principles, including reflective practice, individualized consideration, and creating safe environments for students to practice leadership behaviors.

Develop comprehensive assessment tools: Curriculum designers should implement reliable tools evaluating mentorship quality and students’ leadership competency progression, distinct from clinical performance evaluation, to continuously improve mentorship programs.

Provide policy support for mentors: Policymakers and administrators should formally recognize and incentivize high-quality mentorship through protected time for mentorship activities and incorporation of mentorship effectiveness into faculty promotion and evaluation criteria.

Design culturally sensitive programs: Future mentorship programs should be designed with cultural sensitivity, particularly addressing observed gender differences, to ensure effective support for all students in developing leadership competencies relevant to the Saudi healthcare context.

## Figures and Tables

**Table 1:** Personal Characteristics of Participants (N = 224).

Category	Subcategory	N	%
Age, years	<20	135	60.3
	≥20	89	39.7
Sex	Male	109	48.7
	Female	115	51.3
Marital status	Unmarried	222	99.1
	Married	2	0.9
Study year	Second	39	17.4
	Third	112	50.0
	Fourth	73	32.6
GPAs	≤3.5	52	23.2
	>3.5	172	76.8

**Table 2:** Effectiveness of Mentorship Among Undergraduate Nursing Students (N = 224).

Category	Subcategory	Mean	SD	Strongly Agree/Agree	
				N	%
Mentorship items	My mentor was accessible.	3.41	1.489	166	74.1
	My mentor demonstrated professional integrity.	3.44	1.441	168	75.0
	My mentor demonstrated content expertise in my area of need.	3.50	1.398	172	76.8
	My mentor was approachable.	3.48	1.430	168	75.0
	My mentor was supportive and encouraging.	3.47	1.455	170	75.9
	My mentor provided constructive and useful critiques of my work.	3.43	1.428	172	76.8
	My mentor motivated me to improve my work product.	3.37	1.488	161	71.9
	My mentor helped provide direction and guidance on professional issues (e.g., networking).	3.46	1.423	170	75.9
	My mentor answered my questions satisfactorily (e.g., timely response, clear, comprehensive).	3.43	1.475	170	75.9
	My mentor acknowledged my contributions appropriately (e.g., committee contributions and awards).	3.39	1.451	168	75.0
	My mentor suggested appropriate resources (e.g., experts, electronic contacts, source materials).	3.49	1.411	174	77.7
	My mentor challenged me to extend my abilities (e.g., risk-taking, trying a new professional activity, drafting a section of an article).	3.34	1.455	167	74.5
Total	Total mentorship effectiveness	3.43	1.34	169	75.4

Note. SD, standard deviation.

**Table 3:** Leadership Competencies Among Undergraduate Nursing Students.

Category	Subcategory	Mean	SD	Positive		Negative	
				N	%	N	%
Competencies	Strategic thinking	2.81	1.01	141	62.9	83	37.1
	Emotional intelligence	2.84	1.00	146	65.2	78	34.8
	Impact and influence	2.80	1.01	147	65.6	77	34.4
	Teamwork skills	2.82	1.03	143	63.8	81	36.2
Total	Total competencies	2.82	1.00	145	64.7	79	35.3

Note. SD, standard deviation.

**Table 4:** Correlation Between Leadership Competencies and Mentoring Among Nursing Students (N = 224).

Category	Subcategory	Mentoring	
		ρ-Value	*p*-Value
Competencies	Strategic thinking	0.607 **	<0.001
	Emotional intelligence	0.624 **	<0.001
	Impact and influence	0.570 **	<0.001
	Teamwork skills	0.625 **	<0.001
Total	Total competencies	0.618 **	<0.001

Note. ρ is a nonparametric statistic that measures the strength and direction of rank-based associations between variables. Double asterisks (**) adjacent to ρ values denote statistically significant correlations at the *p* < 0.01 level (two-tailed).

**Table 5:** Association Between Leadership Competencies, Mentoring, and Participant Characteristics (N = 224).

Category	Subcategory	Leadership				Mentoring			
Demographics		Mean	SD	z	*p*	Mean	SD	z	*p*
Age/years	<20	2.79	1.04	−0.487	0.626	3.54	1.37		
	≥20	2.87	0.94			3.27	1.29	−1.834	0.067
Sex	Male	2.60	0.91	−3.956	<0.001	3.01	1.39		
	Female	3.03	1.04			3.84	1.16	−4.467	<0.001
Marital status	Unmarried	2.82	1.00			3.44	1.34		
	Married	2.77	0.33	−0.296	0.767	2.50	0.71	−1.089	0.276
Study year	Second	2.99	1.15	2.778	0.249	3.88	1.49		
	Third	2.77	0.91			3.47	1.31		
	Fourth	2.81	1.04			3.14	1.24	11.092	0.004
GPAs	≤3.5	2.51	1.02	−2.510	0.012	3.27	1.45		
	>3.5	2.91	0.97			3.48	1.30	−0.845	0.398

Note. SD, standard deviation; GPA, grade point average.

**Table 6:** Multiple Linear Regression Analysis with Bootstrapping for Predicting Leadership Competencies (N = 224).

Category	Subcategory	B	Std. Error	β	*t*	*p*	95% Confidence Interval	
							Lower	Upper
Predictors	(Constant)	−0.016	0.485		−0.034	0.973	−0.950	0.944
	Mentoring	0.478	0.048	0.642	11.477	<0.001 **	0.382	0.570
	Sex	−0.004	0.108	−0.002	−0.040	0.968	−0.214	0.209
	Grade	0.126	0.089	0.088	1.635	0.104	−0.046	0.296
	GPAs	0.202	0.083	0.131	2.481	0.014 *	0.043	0.366

Note. BCa, bias-corrected and accelerated confidence intervals based on 5000 bootstrap samples; GPA, grade point average. F (38.089); * *p* < 0.05; ** *p* < 0.001; R (0.641); adjusted R^2^ (0.399).

## Data Availability

All data supporting the findings of this study are provided within the article. No additional datasets were generated or analyzed during the current study; therefore, there are no supplementary data to share publicly. The study was conducted at Northern Border University, and due to institutional policies and confidentiality considerations, raw data cannot be made publicly available.

## Supplementary Materials

The following supporting information can be downloaded at: https://www.mdpi.com/article/10.3390/nursrep16010013/s1, Table S1: Leadership Competencies Among Undergraduate Nursing Students. Table S2: Correlation Between Leadership Competencies and Mentoring Among Nursing Students (N = 224).

## Public Involvement Statement

Nursing students participated in this study by completing a structured survey, providing essential insights into mentorship and leadership development in Saudi nursing education. They were not involved in the study design, analysis, or dissemination. Future research will aim to include students more actively in these stages to enhance relevance and impact.

## Guidelines and Standards Statement

This manuscript was drafted in accordance with the Strengthening the Reporting of Observational Studies in Epidemiology (STROBE) Statement for cross-sectional research [40].
