# Supplementary material for: Mentorship Quality and Leadership Development in Saudi Nursing Education: A Cross-Sectional Analysis
Source: Nurs Rep. 2026 Jan 5;16(1):13. doi: 10.3390/nursrep16010013 (PMC12844347; doi:10.3390/nursrep16010013)
Supplement: Supplementary file 1 [file nursrep-16-00013-s001.zip › Supplemental_Table_S1.pdf]

Supplemental Table S1: Leadership Competencies Among Undergraduate Nursing Students

	Mean	SD	Strongly	
			N	%
Evaluate your own needs	2.80	1.208	141	62.9
Fully grasp the ideas of the problem	2.85	1.161	142	63.4
Are aware of how you communicate with others	2.91	1.164	151	67.4
Are able to persuade groups to agree on specific issues	2.74	1.142	138	61.6
Organise your thoughts clearly and logically	2.89	1.141	146	65.2
Listen attentively for meaning and feelings	2.91	1.133	151	67.4
Get others to work together effectively	2.84	1.172	150	67.0
Predict the consequences of your decisions	2.77	1.190	137	61.2
Aware of the perceptions of others	2.71	1.163	134	59.8
Encourage the understanding of the point of view of other group members	2.75	1.246	135	60.3
Plan ahead for what should be done	2.84	1.163	143	63.8
Recognise and locate resources to resolve a problem	2.84	1.143	146	65.2
Show a willingness to make changes	2.83	1.172	144	64.3
Influence a group in goal-setting	2.82	1.170	147	65.6
Make decisions on a factual basis	2.88	1.205	152	67.9
Alter your own behaviour to meet a situation	2.81	1.196	142	63.4
Strive to understand other people	2.79	1.196	140	62.5
Assume responsibility for action taken based on your own decisions	2.90	1.192	153	68.3
Try to learn what impact you make on others	2.89	1.141	146	65.2
Grasp the essentials of a problem, see solutions, and choose a course of action	2.89	1.156	150	67.0
Hold the attention of others while presenting pertinent ideas	2.79	1.197	143	63.8
Try new ideas in a group	2.75	1.171	133	59.4
Delegate responsibility appropriately	2.66	1.187	125	55.8
Feel good about face-to-face exchanges of ideas	2.79	1.180	142	63.4
Discriminate between relevant, irrelevant, essential, and accidental data	2.79	1.174	144	64.3
Get others to follow your advice and direction	2.83	1.149	145	64.7
Encourage group members to work as a team	2.83	1.154	147	65.6
Direct group members or instruct them on what to do	2.79	1.197	141	62.9
Originate new approaches to problems	2.64	1.163	124	55.4
Have group members share in the decision-making	2.88	1.161	152	67.9
Look for ways to improve yourself	2.91	1.115	148	66.1
Initiate action for new and better procedures and policies	2.82	1.154	140	62.5
Know how to proceed to get something done	2.82	1.192	145	64.7
Are friendly and approachable	2.83	1.219	143	63.8
Stand up for a group even if it makes you unpopular	2.73	1.213	129	57.6
Can define your role in a situation	2.78	1.221	138	61.6
Explain the reason for the criticism	2.79	1.243	140	62.5
Encourage group members to express their ideas and opinions	2.92	1.141	152	67.9
Encourage slow-working members to improve their effort	2.86	1.147	146	65.2
Give credit when credit is due	2.84	1.159	143	63.8

Note. SD, standard deviation.
