# Supplementary material for: Mentorship Quality and Leadership Development in Saudi Nursing Education: A Cross-Sectional Analysis
Source: Nurs Rep. 2026 Jan 5;16(1):13. doi: 10.3390/nursrep16010013 (PMC12844347; doi:10.3390/nursrep16010013)
Supplement: Supplementary file 1 [file nursrep-16-00013-s001.zip › Supplemental_Table_S2.pdf]

Supplemental Table S2: Correlation Between Leadership Competencies and Mentoring Among Nursing Students (N=224)

Variables	Mentoring	
	***ρ*-value**	***p*-value**
Strategic thinking	.607**	<.001
Emotional intelligence	.624**	<.001
Impact and influence	.570**	<.001
Teamwork skills	.625**	<.001
Total competencies of leadership	.618**	<.001

Note. *ρ* is a nonparametric statistic that measures the strength and direction of rank-based associations between variables. Double asterisks (**) adjacent to *ρ* values denote statistically significant correlations at the *p* <.01 level (two-tailed).
